# *Streptococcus dysgalactiae subsp. dysgalactiae* presents with progressive weakness in limbs: a case report and literature review

**DOI:** 10.1186/s12879-023-08190-3

**Published:** 2023-03-30

**Authors:** Chen-Hong He, Shu-Fan Feng, Shu-Xin Chen, Deng-Ke Han, Tian-Rong He, Jian-Wei Cao, Hui-Qiang Mai

**Affiliations:** 1 Department of Emergency, Zhongshan City People’s Hospital, No. 2 of Sun-Wen East Road, Zhongshan, 528403 China; 2 Department of Pediatrics, Zhongshan City People’s Hospital, No. 2 of Sun-Wen East Road, Zhongshan, 528403 China; 3 Department of Laboratory Medicine, Zhongshan People’s Hospital, Zhongshan, 528403 China

**Keywords:** *Streptococcus dysgalactiae subsp. dysgalactiae*, Muscle weakness, Penicillin, Case report

## Abstract

**Background:**

*Streptococcus dysgalactiae subsp. dysgalactiae* has been identified as an animal pathogen that is thought to occur only in animal populations. Between 2009 and 2022, humans infected with SDSD were reported rarely. There is a lack of details on the natural history, clinical features, and management of disease caused by this pathogen. This case outlines a human SDSD with muscle aches and progressive loss of muscle strength leading to immobility and multi-organ dysfunction syndrome.

**Case presentation:**

She presented with muscle pain and weakness, and later developed a sore throat, headache and fever with a maximum temperature of 40.5 °C. The muscle strength of the extremities gradually decreased to grade 1 and the patient was unable to move on his own. Next-generation blood sequencing and multi-culture confirmed the presence of *Streptococcus dysgalactiae* and *Streptococcus dysgalactiae subsp. Dysgalactiae,* respectively. A Sequential Organ Failure Assessment score of 6 indicated septicemia, and therapeutic antibiotics were prescribed empirically. After 19 days of inpatient treatment, the patient's condition greatly improved and completely recovered within a month.

**Conclusion:**

Symptoms of *Streptococcus dysgalactiae subsp. dysgalactiae* presenting with progressive limb weakness resemble polymyositis, so a precise differential diagnosis is essential. Multidisciplinary consultation is helpful when polymyositis cannot be ruled out and facilitates the choice of an optimal treatment protocol. In the context of this case, penicillin is an effective antibiotic for *Streptococcus dysgalactiae subsp. dysgalactiae* infection.

## Background

There are only 12 documented cases of *Streptococcus dysgalactiae subsp. Dysgalactiae* (SDSD) infection in humans, so information characterizing the clinical picture and therapeutic protocol of SDSD is still in the exploratory stage. In this area, case profiles involving multisystem dysfunction syndromes, especially neuromuscular symptoms, have not been well described. We report the first case of SDSD infection identified in our department. This study details the clinical progression of muscle weakness and the course of antibiotic treatment. The case report was written in accordance with the CARE Guidelines [1].

## Case presentation

The patient, a 31-year-old female, presented to our emergency department with complaints of fatigue and muscle pain in both lower extremities for two days, which had worsened a day earlier and manifested difficulties with daily activities. She initially presented to the local hospital with chills, fever, and headache. In response to treatment, the symptoms were temporarily relieved (unavailable medication information). Blood results showed thrombocytopenia and compromised liver function. Given the complexity of her condition, the primary care physician recommended a referral to an advanced hospital. The patient underwent a medication abortion and curettage at 11 weeks’ gestation five days ago. There was no discomfort during the operation or in the postoperative period, except for a very small amount of brown secretion. She had a cesarean section in 2005. There was an absence of exposure to grass or animals recently. It was evident from the physical examination that both lower extremities were tender and that bilateral tonsils were enlarged to a first degree. Vital signs were a temperature of 37.4 °C, a pulse of 119 bpm, 28 breaths per minute, 98% oxygen saturation (in room air), and normal blood pressure. The results reported tachycardia on electrocardiogram, normal echocardiogram, and mild enlargement of the liver on abdominal computed tomography (CT) scan.

By CARE guidelines, the timeline in Table 1 illustrates the chronological sequence of pertinent events in the patient's medical file [1].

**Table 1:** The manifestations, diagnostic testing, and interventions in the patient's medical file

Dates	Relevant past medical history and interventions		
22/3–26/3	Five days ago, she underwent medical abortion and curettage, and later felt muscle pain, fatigue, fever, sore throat, and headache. The local results indicated hypo thrombocytosis and liver function damage and received anti-thermal treatment (unspecified medicine)		
Dates	Summaries from Initial and Follow-up Visits	Diagnostic Testing	Intervention
2022/3/27	Aggravating muscle soreness for two days, followed by chills, fever, sore throat for one day	Lab’s test: Routine blood, specimen culture, Weil-Felix Reaction, Dengue fever antigen and Widal Reaction Chest and Abdomen CT Doppler Ultrasound in Gynecology	1 Empirical doxycycline 0.2 g 2. Symptomatic and supportive treatment 3 Monitor urine output 4 Gynecological Consultation
2022/3/28	Dry mouth and mild edema in lower extremities; blurred vision and pain in both eyes, and conjunctival hyperemia	CRP 200.30 mg/L and Myoglobin 675.90 ng/ml Additional lab examinations include Epidemic hemorrhagic fever antibody and parathyroid hormone etc Mydriatic fundus photography or OCT fundus scan High-throughput sequencing testing	1 Moxifloxacin 0.4 g QD 2 Nephrology and Ophthalmology Consultation 3 A high-quality low-protein diet and compound α-keto acid in three meals 4 Sodium bicarbonate 1.0 g TID 5 Atomolan 1 g*QD;
2022/3/28	Chest tightness, shortness of breath (SOB), decreased urine output, and severe pitting edema in lower extremities at night	Lactic acid: 5.14 mmol/L and Pro-BNP: 3222 pg/ml CVP = 1cmH2O Creatinine 140umol/L and Urea 10.77 mmol/L	1. Indwelling CVC and urinary catheter 2. Diuresis but without urine output, and then fluid resuscitation
2022/3/29	Increased swelling in lower extremities, dry mouth, blurred vision in both eyes, and orbital pain	Labs: CRP 193.60 mg/L; IL-6 4847 pg/ml, PCT 13.11 ng/ml; WBC 13.73*10^9/L, PLT 42*10^9/L; D-D 5.57 mg /L, FDP 2.40 mg/L and hypothyroidism	1 Moxifloxacin 0.4 g QD 2 Symptomatic treatment such as diuretics
2022/3/30	Reduced muscle strength from level 3 to 1	Cerebrospinal fluid workup Further endocrine testing: cortisol, ACTH etc	1 Moxifloxacin 0.4 g QD 2 Neurology and Endocrinology consultation 3 Lumbar punctures
2022/3/31	Unrelieved muscle soreness, Passive position (bedridden)	The total amount of protein testing etc EMG and myositis antibody test, and muscle biopsy if necessary	1 Supplemental albumin therapy and discontinuation of diuretics 2 Glucocorticosteroid therapy is contraindicated
2022/4/1	Improved muscle strength	Increased CK and CK-MB	1 Piperacillin-tazobactam sodium 4.5 g*Q8H 2 Nutritional support 3 Monitor fluid balance
2022/4/3	Fatigue and muscle soreness improved slightly。	24-h urine volume: 4250 ml/C, fluid balance: -2098 ml CVP = 3cmH2O	1 Penicillin 4.8 million U*BID 2 Pharmacist, Rheumatology, and Immunology Consultation 3 Fluid resuscitation and albumin supplementation
2022/4/3	Transient chest tightness and SOB at night	HGB 76 g/L and Pro-BNP: 5182 pg/ml, etc	1 Diuretics
2022/4/4	Edema and muscle tenderness improved but still SOB	Normal CK and CK-MB Chest CT: Slight bilateral pleural effusion, enlarged heart, and minor pericardial effusion	1 Penicillin 4.8 million U*BID 2 Continue albumin therapy, and chest tube drainage if necessary 3 Prevent pressure injuries
2022/4/6	Right eye swelling and pain; Bedsores on the sacrococcygeal region	Ophthalmology Consultation: right eye conjunctival hyperemia	1 Penicillin 4.8 million U*ID 2 Tobramycin Eye Drops 3 Skin care
2022/4/9	Active position and soreness in limbs without tenderness	The upper and lower Muscle Strength grading were 5 and 4, respectively;	Penicillin 4.8 million U*BID
2022/4/11	Walking slowly, mild muscle pain	Midstream urine culture: ESBLs without manifestations Echocardiography: minor pericardial effusion	1 Penicillin 4.8 million U*BID 2 Removed the urinary catheter and CVC on 10/4 and 12/4 respectively
2022/4/13	Muscle soreness, muscle strength level 5	WBC 7.21*10^9/L, HGB 88 g/L, PLT 467*10^9/L; PCT 0.05 ng/ml; Pro-BNP 915.70 pg/ml; creatinine 48umol/L, albumin 35.50 g/L;	Discharged on 14th April and outpatient follow-up
2022/5/18	Recovery		

Gynecologic evaluation revealed a small amount of brown secretion and an enlarged uterus with slight tenderness. Magnetic resonance imaging (MRI) and Magnetic resonance angiography (MRA) of the brain were free of abnormalities. In the cases of human SDSD, clinical documentations are not well defined. The results of multi-specimen cultures were decisive in confirming the diagnosis of SDSD. The application of piperacillin-tazobactam (a broad-spectrum antibiotic) failed to improve the clinical picture. There was a parallel improvement in clinical symptoms and laboratory values with the switch to penicillin, which is effective against streptococci. Differentiation of polymyositis presenting as progressive muscle weakness is necessary because the initial treatment regimen includes high doses of corticosteroids rather than antibiotics. We strongly recommended a muscle biopsy for polymyositis, which was rejected by the patient and her husband for financial reasons.

She complained of dry mouth, blurred vision, orbital pain in both eyes, as well as decreased urine output and hypotension. The ophthalmic examination was unremarkable, and the serum-specific antibody test was negative, hence the epidemic hemorrhagic fever was exonerated. The lumbar puncture procedure revealed clear cerebrospinal fluid and an open pressure of 220 mm Hg. Meanwhile, neurologists helped to identify Guillain-Barré syndrome or other neurological disorders. Because SDSD symptoms overlap with multiple diseases, clinicians need to make the necessary differential diagnosis and select agents depending on illness progression and exam findings. Penicillin is an effective treatment for SDSD infections, but one should be wary of the incidence of multidrug resistance.

It is essential to collect specimens for laboratory culture and identification prior to initiating empirical antibiotic therapy. Antibiotics should be altered according to one’s symptoms, inflammatory markers, and susceptibility testing results. In terms of treatment, she received nutritional support and albumin infusion to correct hypoalbuminemia, as well as multidisciplinary consultation for clinical diagnosis and medication direction. To facilitate the management of her condition, central venous catheters and urinary catheters are inserted to monitor fluid intake and output. It was initially prescribed with Doxycycline Hydrochloride at 0.2 g every 12 h and later switched to Moxifloxacin at 0.4 g daily. Drug sensitivity test confirmed that SDSD was sensitive to penicillin G, vancomycin, linezolid, ampicillin, and levofloxacin, but resistant to clindamycin and tetracycline. Accordingly, an alternative dose of piperacillin-tazobactam sodium 4.5 g every 8 h was administered. There was an improvement in muscle strength with piperacillin-tazobactam sodium, while the fever had not resolved. It was eventually shifted to a dose of 4.8 million units of penicillin twice daily through multidisciplinary consultation and discussion. With nine days of penicillin monotherapy, the condition improved remarkably, and the laboratory values normalized. She was discharged on the 19th day of admission. Table 2 illustrates the relevant laboratory results and antibiotic profiles.

**Table 2:** Antibiotics Administration and Important Laboratory Results

**Items**	DATE	27th Mar	28th	29th	30th	31st	1st April	2nd	3rd	7th	11th	13th	14th
	Antibiotics	D* BID	M* QD	M* QD	M* QD	M* QD	P–T* Q8H	P–T* Q8H	P–T*once + P* BID	P* BID	P* BID		
	**Laboratory Results**												
Cultures	Cervical secretions					SDSD 2 +							
	Midstream Urine					SDSD > 10,000					ESBLs		
	Peripheral Blood					SDSD							
	CSF					Normal							
NGS	Peripheral Blood				SD								
Myositis Antibodies								(-)					
Electromyography								(-)					
LAC (0.60–2.20 mmol/l)			5.14						1.83				
NT-proBNP (0-125 pg/ml)		120	3222	3018					5182	2068		915.7	
IL-6 (0.00 ~ 7.00 pg/ml)		1426		4847					92.03				
PCT (0.00 ~ 0.050)		7.83		13.11		0.07			0.22	0.19		0.05	
hs-CRP (0.0–5.0 mg/L)		218.3				141.7			56.7				
CRP (0.0–5.0 mg/L)		200.3		193.6									
WBC (3.69 ~ 9.16*10^9/L)		6.73		13.73		27.69			16.04	9.33		7.21	
NEUT% (50.0 ~ 70.0%)		88.5		92		93.6			82.3	78.2		68.8	
PLT (101 ~ 320*10^9/L)		37		42		59			218	459		467	
HGB (113-151 g/L)		124		101		79			76	87		88	
Cr(45–84(umol/L)		192	140			84			86			48	
AST (13 ~ 35U/L)		129				125			33	64	29		
ALT(7 ~ 40U/L)		59				41			35	64	46		
ChE(5000-12000U/L)		4344				1397			1577	2154		3153	
CK (41 ~ 186U/L)		533				762							
CK-MB(0-24U/L)		17				78			6				
MYO(0-110 ng/ml)		675.9											
TB-Ab-IgG		(-)											

(1) *:D*: Doxycycline hydrochloride 0.2 g; M*: MoxifloxacinHydrochlorid 0.4 g; P–T*: Piperacillin-Tazobactam 4.5 g; P*: Penicillin 4.8 million U. (2) Normal range values in parentheses

Multidisciplinary consultation and laboratory results helped to discriminate the condition. Neurologists and rheumatologists indicated that myalgia and elevated creatine kinase were signs of multisystem involvement in the setting of severe infection, and multiple cultures were SDSD, further ruling out polymyositis. Endocrinologist confirmed that a lack of endocrine dysfunction affected her muscle strength. In the absence of a history of thyroid disease, hypothyroidism due to T3 syndrome was related to her current underlying disease and hypoproteinemia. For the full treatment period, the patient has not received corticosteroids and recovered completely within one month of follow-up. She developed generalized oedema upon admission, sudden onset of chest tightness and shortness of breath at night, and a significant rise in NT-proBNP. It was noted that the decreased creatinine value remained at a high level, so the sharp increase in NT-proBNP was linked to renal compromise. Moreover, the failure of diuretics to increase urine output, coupled with low CVP, was highly assumed to be multi-organ dysfunction. In a further, there was a pressure injury to the sacrococcygeal skin due to being bedridden for eight days. A final multidrug resistance to extended-spectrum beta-lactamases (ESBLs) produced by Escherichia coli was detected in midstream urine cultures. The retained urinary catheter was considered for possible colonizing bacterial infection due to the absence of symptoms of urinary tract irritation.

**Fig. 1:**
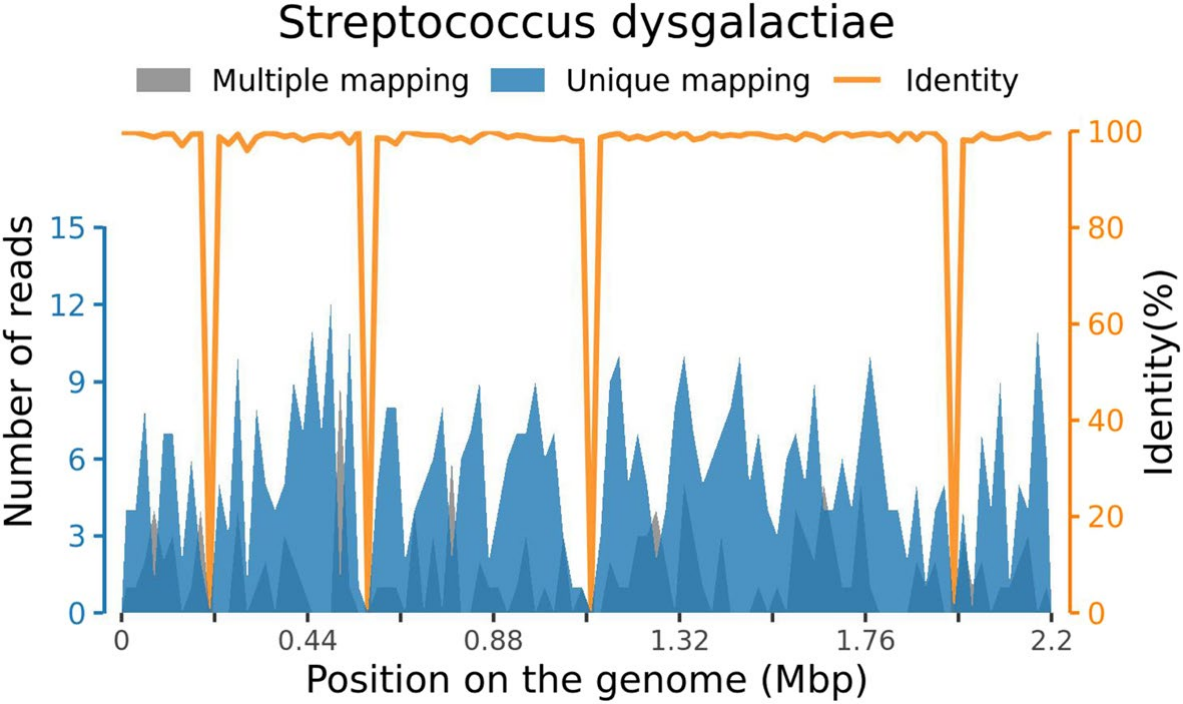
Reads Distribution In The Genome Of The Organisms

There were 534 reads of *Streptococcus dysgalactiae* from peripheral blood detected by comparison with the reference database. The NGS-related information is shown in Table 3 and Fig. 1.

**Table 3:** Metagenomic Testing Report of Pathogenic Organisms IN Peripheral Blood

Quality Control of the Sequencing					
Total reads	Total Base	Q30 Ratio (%)	Quality assessment		
47,862,478	2,393,123,900	95.8	PASS		
SUSPECTED PATHOGENIC ORGANISMS					
Type	Genus	Species	Genus Relative abundance (%)	Genus read number	Species read number
Bacteria, G +	Streptococcus	Streptococcus dysgalactiae	60.63	787	534
SUSPECTED HUMAN MICROBIAL FLORA					
Type	Genus	Species	Genus Relative abundance (%)	Genus read number	Species read number
Bacteria, G +	Staphylococcus	Staphylococcus warneri	0.85	11	5
Bacteria, G +	Staphylococcus	Staphylococcus epidermidis	0.85	11	2
Bacteria, G +	Cutibacterium	Cutibacterium acnes	0.54	7	6
Bacteria, G-	Moraxella	Moraxella osloensis	0.31	4	4

## Discussion

Depending on the hemolytic patterns, *Streptococcus dysgalatiae* (SD) is divided into two subspecies, *Streptococcus dysgalactiae subspecies dysgalactiae* (SDSD) and *Streptococcus dysgalactiae subspecies equisimilis* (SDSE) [2]. A human pathogen (SDSE) that causes various infections similar to Streptococcus pyogenes [3], and an animal pathogen (SDSD) that is thought to occur only in animal populations [4]. A genuine and practical issue of SDSD infections is that it is challenging to identify in laboratory samples. Jensen and Kilian have found that SDSD can be identified as SDSE based on the presence of the β-hemolysis property [5]. In humans [6] and animals [5], both α- and ß-hemolytic isolates have been detected. The blood culture isolates from one patient were α-hemolytic, consistent with the case report by Bansal et al. [7], while isolates from the other one were ß-hemolytic [6]. In a study by Koh et al. of three cases, blood cultures showed weak ß-hemolytic in case 1, β-hemolytic in wound cultures in case 2, and α-hemolytic in blood culture in case 3 [8]. It is likely that the incidence of SDSD human infection is underestimated by the failure to accurately determine the classification of SD subspecies from the hemolytic patterns [9]. To date, the methods employed for SDSD identification in the available case reports include the following, ranging from the 16S rRNA gene [8], a combination of phenotypic features, MALDI-TOF MS (matrix-assisted laser desorption ionization-time of flight mass spectrometry), and 16S rRNA analysis [10], to whole-genomic DNA sequencing [6]. Researchers concluded that the multigene approach is the only way to identify SDSD [6]. Both this study and the two former reports [7, 11] identified the isolate as SDSD through an automated system. Metagenomic next-generation sequencing (mNGS), also known as high-throughput genetic testing, is increasingly being applied to enhance the detection, investigation and diagnosis of infectious diseases [12]. The blood mNGS was sent to a third-party laboratory to gather additional information and it confirmed the presence of *Streptococcus dysgalactiae*. We have not conducted further analysis of this isolate from the results of mNGS, as laboratory tests on the second day confirmed the SDSD. The mNGS as a non-routine test is not reimbursable by Medicare. Patients must bear the cost of expensive tests, which can be a burden in some cases. In fact, mNGS is essential to better understand the pathogenicity and transmission of SDSD. It may be beneficial that genetic analysis will be able to identify factors associated with virulence and host specificity [13].

As shown in Table 4, information on clinical symptoms, detection methods, and antibiotic treatment is provided in case reports of SDSD infections in humans.

**Table 4:** Summary of patients' symptoms, testing methods and antibiotic treatment details from case reports of human infections due to *Streptococcus dysgalactiae subspecies dysgalactiae*

Author and Year	Country	Patient characteristics	Exposure Factors	Complaints/Symptoms	Culture	Testing Methods	Antibiotics Therapy	Outcome
Koh et al.,2009 [8]	Singapore	①67-year-old Chinese Female	seafood	Fever, chills, and rigors with swelling of the right index finger	Blood	16S rRNA gene sequencing	Cloxacillin, penicillin and Gentamicin	Recovered
		②24-year-old Chinese male chef	seafood	The wound required debridement	Wound	16S rRNA gene sequencing	Cloxacillin, Sulphamethoxazole–Trimethoprim and Doxycycline	Recovered
		③48-year-old Chinese Female^a^	seafood	Fever, redness, and pain involving the right breast and arm	Blood	16S rRNA gene sequencing	Ciprofloxacin and Clindamycin (allergy to Cephalosporins)	Recovered
Park et al., 2012 [14]	Korea	61-year Male	Nil	Pain, swelling, burning sensation, and limited range of motion in the right knee	Purulent exudate and synovial fluid	Unavailable	Vancomycin and ceftriaxone	Recovered
Jordal et al., 2015 [10]	Norway	65-year Male	Nil	Fever, radiating pain, muscle ache and inaccuracy of vision	Blood	Combination of phenotypic characteristics, MALDI-TOF MS, and 16S rRNA	Meropenem and Vancomycin; Ceftriaxone and Gentamicin clindamycin	Recovered
Chennapragada et al., 2018 [11]	India	49-year Female	Nil	Fever, pain, redness, swelling and a restricted range of motion	Blood	Automated system	Vancomycin and Cefepime (in hospital) and Ceftriaxone/IV (at home)	Recovered
Im et al., 2019 [15]	Korea	22-day neonate	Nil	Fever, possible visual abnormalities	Cerebrospinal fluid	Unavailable	Initially Ampicillin (300 mg/kg/day) and cefotaxime (200 mg/kg/day); later Ampicillin and Gentamycin for 4 weeks	Recovered
Koh et al.,2020 [6]	Singapore	48-years/Female^a^	seafood	Fever, redness, and pain involving the right breast and arm	Blood	Genomic DNA sequencing	Ciprofloxacin and Clindamycin (allergy to Cephalosporins)	Recovered
		the second patient	unknown	Ipsilateral arm lymphoedema	Blood	Genomic DNA sequencing	Unavailable	Unavailable
		the third patient	poultry	Ipsilateral upper limb cellulitis and oedema	Blood	Genomic DNA sequencing	Unavailable	Unavailable
Bansal et al.,2020 [7]	India	40-years-old Female	suspected an infected animal	Pain in pelvic and suprapubic region	Blood	Automated system	A trial of oral cefixime 400 mg/day for 10 days	Recovered
Nathan et al., 2021 [16]	India	45-year Male	Nil	Swelling of the right upper limb; backache with generalized myalgia	Blood culture	Unavailable	Piperacillin/tazobactam and vancomycin	Died
This study	China	31-year Female	Medical abortion and curettage	Fever, aggravating muscle soreness, chills, sore throat and headache	Cervical secretions, blood, urine	Automated system	Doxycycline; Moxifloxacin Hydrochlorid; Piperacillin-Tazobactam; Penicillin	Recovered

^a^ The same patient in two studies

It is critical to note that the severity of the clinical symptoms of SDSD can range from mild localized pain to severe, life-threatening conditions. Some cases of this pathogen manifesting fever and pain have been reported in the literature [1–7], including two cases with visual abnormalities [3, 5] and one case with generalized myalgia [7]. Currently, three cases of ocular problems associated with SDSD infection result in impaired vision. One case involved a 65-year-old male with inaccurate vision [3], another involved a 22-day-old neonate with a visual path abnormality [15] and the 32-year-old female, in this case, had blurred vision. In terms of laboratory findings, elevated total creatinine kinase (CK) fraction could predict rhabdomyolysis or myositis among patients with cellulitis. However, excessive creatine kinase levels were not expected in patients with cellulitis [7]. The myoglobin level on admission was 675.9 ng/mL (0–110 ng/mL) and the CK level (41–186 U/L) increased from 533 u/L to 762 u/L on day five. With progressive muscle weakness in the extremities, polymyositis may have been present. However, the consensus from the multidisciplinary consultation was that muscle weakening and soreness were associated with sepsis and that polymyositis was not considered. Furthermore, the patient refused a muscle biopsy to confirm the presence of muscle inflammation typical of polymyositis. There was ultimately a lack of clear evidence for the diagnosis of polymyositis. It was conclusively excluded when the patient had recovered to the pre-onset state without further medical intervention. Despite mentioned conditions, SDSD-induced hematological disorders are equally noteworthy, such as the reduction of hemoglobin and platelets 76 g/L and 37*10^9/L, respectively.

Regarding the management of serious infections, effective and immediate intravenous empirical antibiotic therapy may be appropriate in the absence of pathogens. The combination of antibiotics is usually common in practice. Notably, the overuse of antibiotics has contributed to the persistence of antibiotic-resistance genes and the emergence of multi-drug resistance in populations, which poses a threat to the cure of common infections and results in long-term illness and death [17]. Of these patients, all but one died of irreversible septic shock [16], and the others benefited from the antibiotic combination and survived. In these cases, vancomycin and ceftriaxone were the most prescribed antibiotics [10, 11, 14, 16]. This case differs from prior cases of antibiotic combination in that each course of treatment is a single antibiotic. Therefore, it is worthwhile to further investigate whether human SDSD strains require antibiotic combinations. To maximize the effectiveness of treatment, clinicians should correctly and appropriately prescribe antibiotics in line with the laboratory pathogen profile and drug sensitivity data. It is assumed that the treatment of SDSE infections applies to SDSE infections as well [18]. Penicillin G currently remains the first-line agent, followed by second- and third-generation cephalosporins [19]. The resistance profile of human SDSD to tetracycline antibiotics seen in this case is consistent with the resistance of bovine SDSD isolates [20] and the results of Alves-Barroco et al. [21].

## Conclusion

Given the increasing number of pathogens rarely associated with causing human diseases and the proportion of immunocompromised individuals, clinicians need to be aware of the pathogenic potential of such isolates. The pathogenicity of SDSD and its clinical features should empower clinicians to formulate the optimal treatment plan for their patients. Next-generation sequencing technology detects thousands of pathogens, minimizing the incidence of erroneous or omitted identifications. Such technology is now well-established and is widely deployed as a powerful diagnostic tool for pathogens [22].

## Data Availability

The data in the study are all from the clinical medical record system of Zhongshan City People’s Hospital in China. All data generated or analyzed during this study are included in this published article, additional specific data can be obtained from the corresponding author.

## Abbreviations

ACTH: Adrenocorticotropic hormone
ALT: Alanine transaminase
AST: Aspartate transaminase
BID: Twice a day
ChE: Cholinesterase
CK: Creatine kinase
CK-MB: Creatine kinase—MB
Cr: Creatinine
CRP: C-reactive protein
CT: Computed Tomography
CVC: Central venous catheter
CVP: Central venous pressure
D-D: D-Dimer
ECG: Electrocardiogram
EMG: Electromyography
ESBLs: Extended-spectrum β-lactams
FDP: Fibrinogen Degradation Product
HGB: Hemoglobin
hs-CRP: High sensitivity C-reactive protein
IL-6: Interleukin-6
IV: Intravenous
LAC: Lactic acid
MALDI-TOF MS: Matrix-assisted laser desorption ionization-time of flight mass spectrometry
mNGS: Metagenomic Next-Generation Sequencing
MRA: Magnetic resonance angiography
MRI: Magnetic resonance imaging
MYO: Myoglobin
NT-proBNP: N-terminal B-type Natriuretic Peptide
OCT: Optical Coherence Tomography
PCT: Procalcitonin
PLT: Platelet
Q8H: Every eight hours
QD: Once a day
SD: Streptococcus dysgalatiae
SDSD: *Streptococcus* *dysgalactiae* *subsp.* *dysgalactiae*
SOB: Shortness of Breath
SOFA: Sequential Organ Failure Assessment
TB-Ab-IgG: Tuberculosis IgG antibodies
TID: Three times a day
WBC: White Blood Cell
